# Draft Genome Sequence of Psychrobacter okhotskensis Strain 5179-1A, Isolated from a Raw Cured Ham Storage Crate

**DOI:** 10.1128/MRA.00682-20

**Published:** 2020-07-16

**Authors:** Joseph Wambui, Marina Morach, Nicole Cernela, Marc J. A. Stevens, Giovanni Ghielmetti, Roger Stephan

**Affiliations:** a Institute for Food Safety and Hygiene, Vetsuisse Faculty, University of Zurich, Zurich, Switzerland; Georgia Institute of Technology

## Abstract

We present the draft genome sequence of Psychrobacter okhotskensis strain 5179-1A, which was isolated from a raw cured ham storage crate. Its size and GC content are 3.4 Mb and 43.4%, respectively. The 16S rRNA sequences of strain 5179-1A and *P. okhotskensis* MD17^T^ are 100% identical.

## ANNOUNCEMENT

Psychrobacter okhotskensis is a facultatively psychrophilic Gram-negative, catalase- and oxidase-positive, nonmotile, aerobic coccobacillus (1). To the best of our knowledge, P. okhotskensis has so far been isolated only from permafrost and the marine environment (1–3), and there is no publicly available genome. Here, we have determined the draft genome sequence of P. okhotskensis 5179-1A.

An inoculum, prepared from a swab sample taken from a crate that was used for storing raw cured ham in Zurich, Switzerland, was streaked onto Columbia blood agar supplemented with 5% defibrinated sheep blood and incubated aerobically at 30°C for 3 days. This strain was purified by subculturing on the same medium under similar growth conditions. Genomic DNA was isolated from the pure culture using the DNA blood and tissue kit (Qiagen, Hombrechtikon, Switzerland). The DNA was prepared using a Nextera DNA Flex sample preparation kit (Illumina, San Diego, CA, USA), and the resulting transposome-based libraries were sequenced on a MiniSeq sequencer (Illumina). The sequencing output was 254 Mb of 150-bp paired-end reads. Reads were checked for quality using the software package FastQC v0.11.7 (4) and then assembled using the SPAdes v3.0 (5)-based software Shovill v1.0.4 (https://github.com/tseemann/shovill). The assembly was filtered, retaining contigs of >500 bp. The genome was annotated using the NCBI Prokaryotic Genome Annotation Pipeline (PGAP) (6) and the RAST pipeline (7). Default parameters were used for all software and Web servers. The sequenced genome was assembled into 72 contigs and comprises 3.4 Mb (GC content, 43.4%) with 2,982 predicted protein-coding sequences. The genome coverage, contig *N*_50_, and contig *L*_50_ were 80.0×, 111,840 bp, and 10, respectively.

RASTtk (7) and tRNAscan-SE (8) identified 47 RNAs and 43 tRNAs, respectively. Species identification was carried out using the 16S rRNA gene-based identification server (9). The strain’s 1,539-bp 16S rRNA sequence was 100% similar, with a coverage of 100%, to that of P. okhotskensis MD17^T^. The GC content of P. okhotskensis 5179-1A is lower than that of P. okhotskensis MD17^T^, which is 46.7% (1). RASTtk (7) identified 24 features for fluoroquinolone, β-lactam, cadmium, copper, zinc, and cobalt resistance or tolerance. VFDB 2019 (10) predicted 22 potential virulence factors, including hemolysin and a siderophore orthologous to Baumannii acinetobactin utilization A protein (BauA). The probability of P. okhotskensis being a human pathogen was predicted to be 0.54 by PathogenFinder (11); 10 pathogenic families were matched from Psychrobacter arcticus 273-4, which corresponds with previous reports that *Psychrobacter* spp. can be considered opportunistic pathogens in humans and livestock (12–15).

### Data availability.

This whole-genome shotgun project has been deposited in DDBJ/ENA/GenBank under the accession no. JABRVQ000000000. The version described in this paper is version JABRVQ010000000. The raw sequencing reads have been deposited in the SRA under the accession no. SRP265202.
